# Impact of vitamin D on gene expression in Atlantic salmon skin

**DOI:** 10.1371/journal.pone.0349197

**Published:** 2026-05-18

**Authors:** Lewis D. Taylor, Courtney E. Gorman, Fintan Egan, Neil M. Ruane, Philip McGinnity, C. Darrin Hulsey

**Affiliations:** 1 School of Biology and Environmental Science, University College Dublin, Belfield, Dublin, Ireland; 2 Marine Institute, Newport, Co. Mayo, Ireland; 3 School of Biological, Earth and Environmental Sciences, University College Cork, Cork, Ireland; Mansoura University, EGYPT

## Abstract

Vitamin D is a crucial micronutrient for vertebrate health that affects musculoskeletal function and likely modulates immune responses in tissues such as the skin. Infectious diseases are a critical concern in Atlantic salmon aquaculture due to the potential economic impact of stock losses, risks to fish welfare, and environmental sustainability. Since the skin is the first line of immunological defence, its ability to mediate host-pathogen interactions may be influenced by dietary vitamin D. This study examined the transcriptomic response of salmon skin after six months of dietary vitamin D supplementation, with an emphasis on immune-related gene expression. A total of 151 differentially expressed genes were identified, 27 of which are linked to inflammation, antigen presentation, and both innate and adaptive immunity. These results indicate that vitamin D could modulate cutaneous immune responses by reducing inflammation and enhancing innate defences, potentially improving resistance to many skin-associated salmon pathogens.

## Introduction

The skin serves as both a defensive barrier and a vital interface between an organism and its environment. In humans, the skin often forms the first line of immunological defence against pathogens and also facilitates the production of the essential nutrient vitamin D_3_ (cholecalciferol) in response to solar UV radiation [1–3]. However, at northern latitudes, limited sunlight during winter restricts natural vitamin D synthesis, necessitating dietary intake from vitamin D-rich sources such as Atlantic salmon (*Salmo salar*) [4,5]. Atlantic salmon are among the richest natural sources of vitamin D [6], and like humans, the vitamin D levels of these fishes fluctuate in response to environmental factors [7–9]. This makes Atlantic salmon ideal candidates for biofortification, the process of enhancing the nutrient content of a food resource through dietary supplementation in aquaculture [8–10]. Vitamin D biofortification of salmon could substantially change skin gene expression and its immunological defence against pathogens.

Vitamin D plays a critical role in health and modulates immune responses in many vertebrates [11]. Vitamin D deficiency is well-known for inducing long-term conditions like rickets, or the softening and weakening of bones, in a wide range of animals like birds, reptiles, ungulates, carnivores, and primates [12–17]. However, in addition to its direct impacts on the skeletal system, vitamin D deficiency is linked to a number of short-term health conditions. In terrestrial vertebrates, vitamin D can mediate respiratory infections [15,18], inflammatory skin disorders [19], and susceptibility to parasites like head lice [20] and leishmaniasis [21]. In some fish, like rainbow trout (*Oncorhynchus mykiss*), insufficient vitamin D is known to weaken skin integrity [22], increasing susceptibility to damage and infection [23]. Importantly, vitamin D could enhance the immune responses of the skin of Atlantic salmon both in freshwater and saltwater environments [24]. The immune responses of farmed Atlantic salmon is of wide interest because these fishes are highly susceptible to several skin pathogens and vitamin D could alter immune gene expression and beneficially mitigate many salmon host-pathogen interactions [24,25].

Vitamin D’s immunomodulatory effects are largely driven by its regulation of immune-related gene expression [11,12]. These genes often fall into major functional categories, including innate and adaptive immunity, inflammation, and antigen presentation [26,27]. In mammals, vitamin D regulates genes involved in innate immune responses that provide immediate but broad-spectrum protection from pathogens [27,28]. Additionally, vitamin D influences adaptive immune responses, which are slower to activate but highly pathogen-specific [29–31]. For example, vitamin D enhances the expression of genes responsible for lysozyme production as well as anti-microbial peptides (AMPs) that are key factors in both innate immunity and antigen presentation [30,32,33]. Additionally, vitamin D regulates inflammatory processes by preventing excessive or chronic immune activation [34,35] and regulating antigen presentation via MHCI (Major Histocompatibility Complex I) [36,37]. While these effects have been studied extensively in mammals, vitamin D’s influence on immune gene expression in fish, such as Atlantic salmon, remains poorly understood.

This study investigates how vitamin D biofortification affects gene expression in Atlantic salmon skin. First, RNA-seq was employed to examine how six months of vitamin D diet augmentation influences general gene expression patterns in Atlantic salmon skin. Then, the study explored whether vitamin D modulates the expression of individual genes and gene pathways associated with immune function.

## Materials and methods

### Experimental populations

Atlantic salmon were raised at the Marine Institute’s freshwater rearing facility in Newport, County Mayo, in Ireland according to detailed methods described previously in Gorman *et al.* [9]. Briefly, two experimental populations were established, one from eggs obtained from a Norwegian commercially farmed strain, the other using eggs acquired from a captive-bred Irish strain used for experimental ocean release (sea ranching) programmes. At the commencement of the first feeding (April 2023), the period when alevins transition from utilising yolk sac reserves to exogenous food, the fry were fed a standard commercial feed for one week before the experimental groups were transitioned to experimental feeds. The Atlantic salmon used in this experiment were monitored daily by research staff trained in animal husbandry and euthanasia. The use of salmon in this study was authorised by The University College Dublin Animal Research Ethics Committee: Research Ethics Reference Number AREC-23-01-Hulsey.

### Vitamin D experimental feeds

The composition of the experimental diets is given in detail in Gorman *et al.* [9]. In brief, to make the experimental diets, we started with a commercial salmon feed that included a small baseline amount of vitamin D. This standard commercial feed served as the control (low vitamin D) diet. Then, to make the high vitamin D diet, we added additional vitamin D_3_ (cholecalciferol) to the commercial feed. This resulted in two experimental diets: 1) low, which contained 866 µg/kg vitamin D_3_, and 2) high, which contained 1779 µg/kg vitamin D_3_. Both specific vitamin D_3_ concentrations were determined using liquid chromatography and mass spectrometry [9]. Both feeds had a basal composition of 50% crude protein and 21% crude lipid, differing only in the concentration of vitamin D_3_. The two diets were provided *ad libitum* to the respective tanks containing the developing salmon.

### Atlantic salmon sampling

The skin of the Atlantic salmon was sampled the week beginning October 1st, 2023, after the fish had been fed experimental diets for approximately six months [9]. At that point, they were sacrificed with an overdose of tricaine methanesulfate (MS-222). Note that the fish were allowed to swim freely in the enclosure before being sacrificed, and none of the fish were euthanised before reaching the experiment’s endpoint. By allowing the fish to swim freely, this reduced the potential stress and suffering when sacrificing the fish. Twelve total individual skin samples were dissected from the dorsal right side of the fish, with six individuals dissected from each vitamin D treatment group. To sample the skin, a rectangular area of approximately 3 x 1 cm was removed, and the skin was then separated from the underlying muscle using a scalpel. The dissected skin samples were stored in RNAlater (Sigma-Aldrich) in 1.5 ml Eppendorf tubes before being sent for RNA sequencing.

### Atlantic salmon skin mRNA sequencing

**Fig 1:**
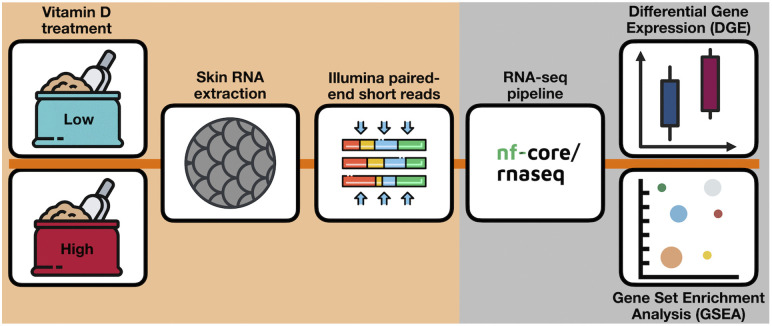
Experimental workflow. The Atlantic salmon RNA-seq data were derived from a previous experiment focused on vitamin D supplementation of salmon [9]. Data was analysed using the Nextflow bioinformatic pipeline [38]. Gene set enrichment analyses (GSEA) were performed for the vitamin D dataset using the clusterProfiler package (version 3.22) in R [39].

To generate RNA-seq libraries for the skin samples (Fig 1), the 12 samples were sent on dry ice to Novogene at their Cambridge Sequencing Centre (Cambridge, UK). Novogene conducted RNA extraction, mRNA isolation, RNA-seq library preparation, and sequencing. The initial raw data produced (fastq) was processed by Novogene using their in-house pipeline. Clean reads were obtained by removing any reads that contained poly-Ns and exhibited signs of low quality. All downstream analyses used high-quality reads. The Atlantic salmon (Ssal_v3.1) was used as the reference genome and annotation model.

### Differential gene expression – vitamin D (low versus high)

To identify genes showing differential gene expression (DGE) between the low and high vitamin D salmon skin, the fastq files produced from Novogene were run through the Nextflow rnaseq pipeline (23.04.3) using nf-core (3.11.2) [38]. Gene-level read counts produced by the pipeline were imported into R using the tximport (version 3.19) package [40]. Differential expression analysis of gene counts was conducted with the DESeq2 R package 1.20.0 [41]. The data set was prefiltered to remove loci with fewer than 10 counts and those detected in fewer than five samples of the total six samples per group to minimise the number of low and zero-count genes. The resulting *p* values were adjusted using Benjamini and Hochberg’s approach to controlling the false discovery rate (FDR) [42]. Genes with an adjusted *p* value less than or equal to 0.05 and with an absolute log_2_ fold change greater than or equal to one found by DESeq2 were assigned as differentially expressed. The batch effect was accounted for between the strains within DESeq2. Differentially expressed genes identified from vitamin D treatment groups were annotated from Ensembl identifiers using the biomaRt package from the ssalar_gene_ensembl database [43].

### Identification of immune genes

Differentially expressed genes from the vitamin D experiment were annotated in several ways to determine if they likely had an immune function. The DGE genes were first annotated using the AnnotationDbi (3.19) package from [44] Bioconductor version 1.66.0 [44]. Genes without annotated Ensembl IDs were subjected to a BLAST [45] search of the longest exon to determine if there was a match on NCBI. Any genes that did not initially have gene symbols on Ensembl but found substantial matches or homologues on BLAST were annotated to further determine their putative function. The matching percentage was set at a minimum of 90% and coverage of 90%. Genes that had names and possessed an accession number were then searched using Web of Science with search terms [gene symbol] AND Immune*. The complete list of annotated genes is provided (S1 Table), but the genes associated with immunity are primarily the focus of our discussion below. Finally, immune gene categories were determined using Web of Science using the individual gene names and the key phrases ‘innate immune system’, ‘adaptive immune system’, ‘inflammation’, and ‘antigen presentation’.

### Gene set enrichment analysis

Gene set enrichment analysis (GSEA) was performed for the vitamin D dataset using the clusterProfiler package (version 3.22) in R [39]. Importantly, this analysis focuses on more than genes identified in the DGE analysis. The full DESeq2 output for all genes was used as input for GSEA, which aggregates per-gene statistics across predefined gene sets, enabling the detection of coordinated yet modest expression changes within functional pathways. GSEA takes the full ranked list of genes that are sorted by log_2_ fold change, and the analysis is weighted by the highest and lowest expressed genes. The latest Atlantic salmon genome annotation (orgDB: Ssal.db accession no. AH119615) was retrieved from the AnnotationHub package (version 3.21) and used alongside the GO.db package [46,47] to acquire Gene Ontology (GO) term mappings. This annotation offers species-specific links between Atlantic salmon gene identifiers and GO categories. The GSEA was conducted on all three GO ontologies, Biological Process (BP), Molecular Function (MF) and Cellular Component. The output includes the enriched pathways, the enrichment fold scores, as well as the genes associated with the enriched terms.

### Statistical analysis

Differential gene expression analyses were performed using the DESeq2 R package [41]. For each gene, a model was fitted to test for differential expression between the two experimental conditions. *P* values were adjusted for multiple testing using Benjamini and Hochberg’s approach to control for the false discovery rate [42]. Genes with an adjusted *p* value less than or equal to 0.05 and an absolute log_2_ fold change of greater than or equal to 1 were considered differentially expressed. Gene set enrichment analyses (GSEA) were performed using the clusterProfiler package in R [48,49]. For the GSEA, significance was determined by an adjusted *p* value of ≤ 0.05 and *q* value ≤ 0.05. The *q* value estimates the positive false discovery rate, which considers the large number of genes used in GSEA when determining the significance of enriched pathways. All code used can be provided upon request.

## Results

Once cleaned, the RNAseq libraries from the high (n = 6) and low (n = 6) vitamin D groups had a mean size of 13.1 Gb and ranged from 10.38 to 16.17 Gb (S2 Table). The mean total mapping rate of the libraries was 86.46% and ranged from 71.35% to 91.51% (S2 Table).

**Fig 2:**
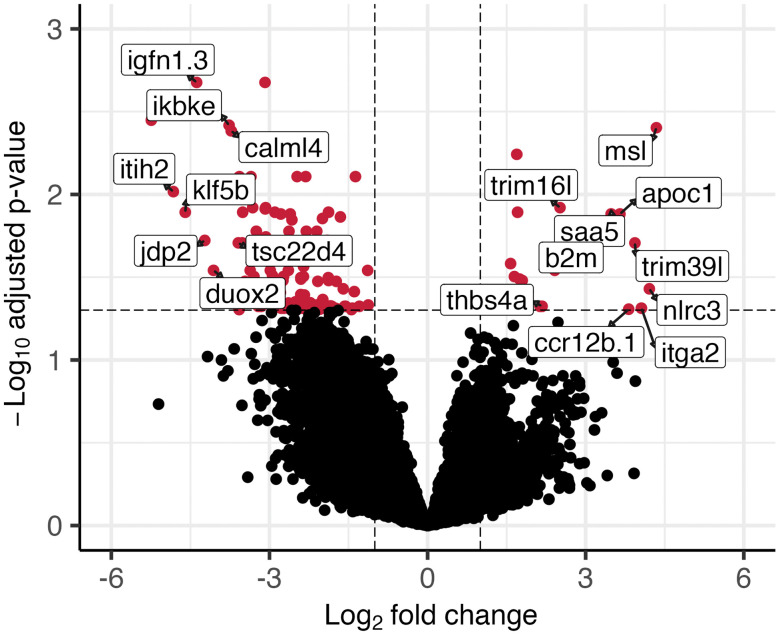
Volcano plot of differentially expressed genes between the high-treatment and low-treatment vitamin D groups. Genes with an adjusted p value less than or equal to 0.05 and an absolute log_2_ fold change greater than or equal to one are shown in red. The labelled points represent annotated genes with the greatest identifiable expression differences.

Of the total 33195 genes inferred to be expressed, 151 genes were found to be significantly differently expressed (adjusted *p* value ≤ 0.05) between the low and high vitamin D treatment groups (Fig 2) in the skin. Of the significant genes, 131 were downregulated, and 20 were upregulated.

The genes with the greatest up- and downregulation were *msl* (mellein synthase-like) with a log_2_ fold change of 4.34 and *lncrna106412* (long non-coding RNA) (log_2_ fold change −7.02) (S1 Table). The differentially expressed genes are involved in a range of functions. For example, *jdp2* (jun dimerisation protein 2) and *calml4* (calmodulin-like 4) are associated with stress and likely involved in calcium signalling (Fig 2) [50,51].

Of the 151 differentially expressed genes, 27 were identified as putative immune genes (Table 1). To facilitate comparisons, immune-related genes were grouped into four key functional categories: innate immunity, adaptive immunity, inflammation, and antigen presentation. Concerning innate immunity, genes such as *bpifcl* (bactericidal/permeability-increasing fold containing family C, like), *cats* (cathepsin S), and *lysc2* (lysozyme)*,* which are involved in antimicrobial defence and lysozyme activity, were upregulated. There was also the upregulation of *cfh* (complement factor H) and downregulation of *c8a* (complement C8 alpha chain), which are both components of the complement cascade. Within adaptive immunity, an upregulation of *ccr12b.1* (chemokine (C-C motif) receptor 12b) and a downregulation of *fcgr1* (FYN-binding protein 1) were observed. These genes are associated with cytokine, immune cell trafficking, and T cell receptor signalling. In the inflammation category, vitamin D supplementation was associated with the downregulation of several pro-inflammatory genes such as *fkbp5* (FKBP prolyl isomerase 5) and *ptgs2a* (prostaglandin-endoperoxide synthase 2a) (S4 Table). Additionally, there was downregulation of *tfa* (transferrin a), which functions as a Fe² ⁺ transporter, and upregulation of antigen presentation gene *b2m* (beta-2-microglobulin).

**Table 1:** Expression of immune-related genes in Atlantic salmon skin following vitamin D supplementation.

Immune Category	Gene Symbol	LFC	Reference
**Upregulated**			
Innate immune system	*apoc1*	3.65	[52]
Innate immune system	*saa5*	3.48	[53]
Innate immune system	*cfh*	1.90	[52]
Innate immune system	*lysc2*	1.71	[54]
Innate immune system	*bpifcl*	1.65	[55]
Innate immune system	*cats-70942*	1.58	[56]
Innate immune system	*cats-10327*	1.24	[56]
Antigen presentation	*b2m*	2.41	[57]
Adaptive immune system	*ccr12b.1*	3.82	[58]
Adaptive immune system	*fcgr1*	1.58	[59]
**Downregulated**			
Innate immune system	*gps1*	−2.29	[46]
Innate immune system	*slc43a3*	−2.63	[60]
Innate immune system	*il31ra*	−2.79	[39]
Innate immune system	*c8a*	−3.07	[61]
Innate immune system	*serpine1*	−3.15	[62]
Innate immune system	*tfa*	−4.84	[47]
Innate immune system	*ikbke*	−3.77	[63]
Inflammation	*fkbp5*	−1.37	[64]
Inflammation	*hspa12b*	−1.43	[65]
Inflammation	*ccl20*	−1.88	[66]
Inflammation	*hsp70*	−2.35	[67]
Inflammation	*hsp70a*	−2.64	[68]
Inflammation	*ccnl1*	−2.90	[69]
Inflammation	*ptgs2a*	−3.37	[70]
Antigen presentation	*clec10a*	−1.14	[71]
Adaptive immune system	*fyb1*	−1.44	[72]
Adaptive immune system	*pak3*	−2.15	[73]

The table displays the log₂ expression levels of representative immune-related genes in the skin of Atlantic salmon treated high doses of vitamin D. Based on the literature, the genes are grouped into four major functional categories: *Innate immunity* upregulated: *bpifcl*, *lysc2*, *saa5*, *cats-70942*, *cats-10327*, *cfh*, and *apoc1* and downregulated: *gps1*, *il31ra*, *serpine1*, *c8a*, *slc43a3*, *tfa*, and *ikbke*. There were no upregulated genes in the inflammation category, but several were downregulated: *hsp70*, *ptgs2a*, *ccnl1*, *hsp70a*, *ccl20,* and *hspa12b*. Antigen presentation upregulated: *b2m* and downregulated: *clec10a* *(loc106567873)*. *Adaptive immunity* upregulated: *fcgr1* and *ccr12b.1*; downregulated: *pak3* and *fyb1*.

**Fig 3:**
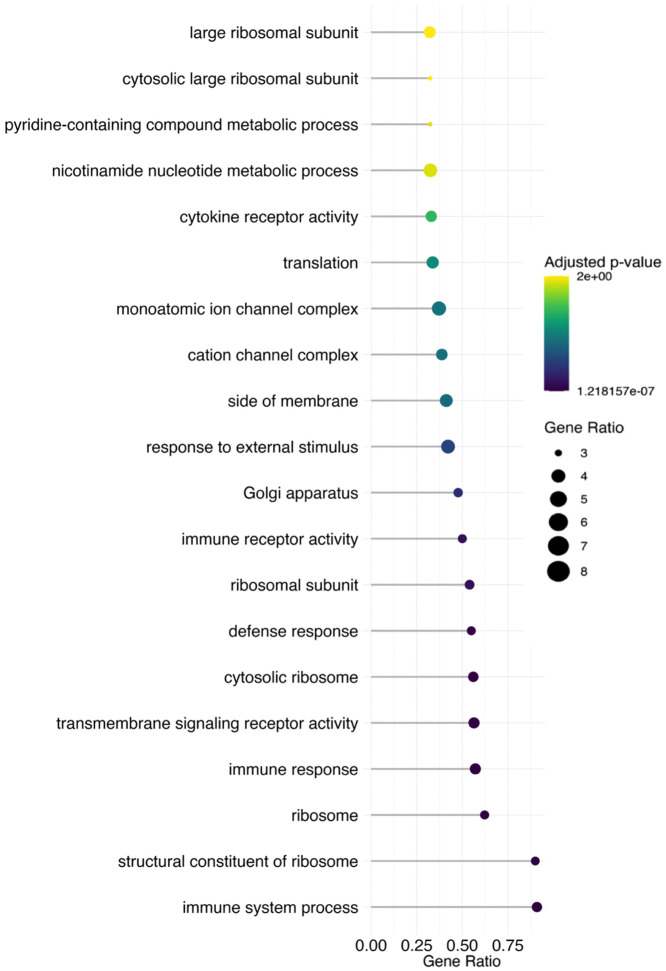
Gene Set Enrichment Analysis (GSEA) of significant genes in the skin between high and low vitamin D treatment. A dot plot displays the top 20 significantly enriched Gene Ontology (GO) terms for downregulated genes, including terms related to enzyme inhibitor activity, negative regulation of peptidase activity, and immune system processes. The size of the dots represents the gene ratio, while the colour gradient reflects the adjusted p value, with shades ranging from green to purple, indicating increasing significance.

In our GSEA of the Atlantic salmon skin in response to vitamin D, we found 126 GO categories that were enriched. For the vitamin D-treated Atlantic salmon, the enrichment analysis found there were 85 matches for biological processes, 22 for molecular function, and 19 for cellular components (Fig 3). The GO category with the greatest gene count was intracellular vesicle. Immune receptor activity was found to have the greatest normalised enrichment score. Several categories associated with the immune system were enriched (S3 Table).

## Discussion

Vitamin D impacts gene expression in the skin of Atlantic salmon. Vitamin D manipulation resulted in 151 differentially expressed genes in the skin (Fig 2), several of which were involved in metabolism and cellular communication. The vitamin D treatments also impacted gene expression in the skin that could broadly affect immune functions such as innate immunity, adaptive immunity, inflammation, and antigen presentation [27]. Of the differentially expressed genes that were detected in response to vitamin D enhancement, 27 of the genes are known to be associated with various aspects of immunity that synergistically could help Atlantic salmon fight pathogens (Table 1). These findings suggest that vitamin D may commonly modulate immune gene expression in Atlantic salmon skin and could play a role in mediating host-pathogen interactions.

Several genes that were differentially expressed in the skin in response to increased vitamin D have broad physiological effects (Fig 3). We found that vitamin D influenced genes such as *serpine1,* which is important for epithelial tissues like skin and the linings of organs and blood vessels, and *calml4,* which is involved in calcium signalling [74,75]. These results may reflect broad effects of vitamin D on general health in salmon as it does in other vertebrates. The similarities and differences between vitamin D’s effects in salmon and other vertebrates would be interesting to investigate further.

Of the differentially expressed immune genes identified, at least 17 have a prominent function in innate immunity, which acts as the first line of pathogen defence by responding rapidly to antigen-independent foreign bodies [75]. For instance, the upregulation of genes encoding lysozyme (*lysc2*) and cathepsin S (*cats*) highlights vitamin D’s role in bolstering innate immunity (Table 1). Lysozyme activity, which can be enhanced by *lysc2* expression, improves defences against bacteria [54]. Increased lysozyme activity in response to vitamin D supplementation has also been observed in other teleost fishes [54]. Additionally, changes were observed in the expression of three genes commonly associated with antimicrobial peptides (AMPs): *saa5, bpifcl,* and *apoc1* (Table 1). The *saa5* gene recruits immune cells to sites of inflammation, aids in identifying pathogens during infections, and can prevent secondary bacterial infections at sites of tissue trauma [76]. The *bpifcl* gene is an AMP that specifically targets gram-negative bacteria, while *apoc1* is known for its bactericidal and defensive role against protozoan ectoparasites [77]. Notably, we did not observe differential expression of cathelicidin, an AMP commonly associated with parasitic defence in Atlantic salmon [78]. However, vitamin D-associated changes in other AMP expression could be important for how Atlantic salmon respond to skin-associated pathogens.

Differential expression of the innate immune genes *cfh* and *c8a* was also observed, and these genes are associated with the complement system [52,61]. Complement is a key component of the innate immune response against invading pathogens and kills pathogens via cytolysis [61]. The complement system is also a vital interface between the innate and adaptive immune system [52,79]. The downregulation of *c8a* in response to higher vitamin D may function to reduce alternative complement activity, prevent excessive inflammation, and ensure targeted removal of pathogens and infected cells [61,79].

Vitamin D may also influence Atlantic salmon’s adaptive immune function [13,32]. Although the majority of genes were downregulated by vitamin D, several upregulated genes likely influence adaptive immune components such as B cells, T cells and antigen-presenting cells [80]. For instance, we observed the upregulation of *fcgr1* (high-affinity immunoglobulin gamma Fc receptor I) alongside the downregulation of *fyb1* (Table 1). These genes are integral to the structure and function of antibodies [52,81–83]. These results underscore the interactions between vitamin D and the adaptive immune response, which warrants further investigation with respect to salmonid host defence.

Vitamin D could also influence how antigens are processed and presented to immune cells [84]. Two of the immune genes identified, *b2m* and *clec10a,* are involved in antigen presentation as part of the MHCI (Major Histocompatibility Complex I) [71,85]. The upregulation of *b2m* may be associated with increased antibacterial activity [85]. In comparison, *clec10a* regulates macrophages in the skin and in mice, interacts with vitamin D [86]. Vitamin D could commonly influence how antigens are presented to the MHCI in the skin and further bolster the skin’s role as the first line of immune defence.

Several differentially expressed genes in the vitamin D treatments could also link innate and adaptive immunity to inflammation. For instance, vitamin D supplementation was associated with the downregulation of pro-inflammatory genes, such as *fkbp5, hsp70*, and *ptgs2a* (Table 1). The *fkbp5* gene functions generally as an immunophilin and plays an important role in innate immune regulation [84]. The *fkbp5* gene is critical for the attachment and internalisation of viruses such as infectious Atlantic salmon anaemia virus (ISAV) in Atlantic salmon cells [87]. Reduced *fkbp5* activity lowers ISAV viral loads [88]. The reduced *ptgs2a* activity, which encodes prostaglandin-endoperoxide synthase 2, is commonly associated with increased vitamin D levels [35,89,90], and in salmonids, *ptgs2a* is an indicator of stress and wound healing [91]. The increased activity of *saa5* in the high vitamin D-treated group may similarly indicate increased anti-bacterial biomarker activity [92]. Vitamin D supplementation could commonly reduce inflammation and bolster immune responses against bacterial infections [64,70].

Our GSEA results (Fig. 3) further highlight a number of responses to vitamin D including the modification of immune and cytokine activity within the skin in response to vitamin D augmentation. This increase in immune activity is in line with differential expression of single genes. Future research into the role of vitamin D in altering Atlantic salmon skin would benefit from direct tests of whether these changes influence the responses to various pathogens. These functional tests of vitamin D fortified salmon could provide additional insight into vitamin D’s role in the immune response and whether the influence is systemic or localised to sites of infection in the skin.

In general, Atlantic salmon biofortification with vitamin D in aquaculture could have multiple beneficial outcomes linked to gene expression changes in the skin. It is now clear that vitamin D biofortification can readily enhance the vitamin D content in fish farmed for human consumption [8,9,23]. Additionally, vitamin D augmentation could bolster the immune responses of Atlantic salmon skin to several pathogens. The addition of greater amounts of vitamin D to Atlantic salmon feed could be a win/win for both human consumption and the skin-associated immune health of Atlantic salmon in aquaculture.

## Supporting information

S1 Table Differentially expressed genes in vitamin D-treated salmon. The table includes the Ensembl ID, log_2_ fold change, adjusted *p* values, and gene symbols. All annotated genes’ accession numbers are from BLAST.
(XLSX)

S2 Table Sample data for RNA-seq output. The table provides information on raw and clean read counts, base counts, sequencing error rates, quality scores (Q20 and Q30), and GC content for each sample.
(XLSX)

S3 Table Gene set enrichment analysis of vitamin D-treated salmon. Includes all outputs from GSEA with significance levels of adjusted *p* ≤ 0.05 and q value ≤ 0.05. The table covers all ontologies related to biological process, molecular function, and cellular component. The table also contains the enrichment and normalised enrichment scores and the set size, which is of genes connected to each GO term.
(XLSX)

S1 File R Code. (ZIP)
